# BCR-Net: A deep learning framework to predict breast cancer recurrence from histopathology images

**DOI:** 10.1371/journal.pone.0283562

**Published:** 2023-04-04

**Authors:** Ziyu Su, Muhammad Khalid Khan Niazi, Thomas E. Tavolara, Shuo Niu, Gary H. Tozbikian, Robert Wesolowski, Metin N. Gurcan

**Affiliations:** 1 Center for Biomedical Informatics, Wake Forest School of Medicine, Winston-Salem, North Carolina, United States of America; 2 Department of Pathology, Wake Forest University School of Medicine, Winston-Salem, North Carolina, United States of America; 3 Department of Pathology, The Ohio State University, Columbus, Ohio, United States of America; 4 Comprehensive Cancer Center, The Ohio State University College of Medicine, Columbus, Ohio, United States of America; Anhui University, CANADA

## Abstract

Breast cancer is the most common malignancy in women, with over 40,000 deaths annually in the United States alone. Clinicians often rely on the breast cancer recurrence score, Oncotype DX (ODX), for risk stratification of breast cancer patients, by using ODX as a guide for personalized therapy. However, ODX and similar gene assays are expensive, time-consuming, and tissue destructive. Therefore, developing an AI-based ODX prediction model that identifies patients who will benefit from chemotherapy in the same way that ODX does would give a low-cost alternative to the genomic test. To overcome this problem, we developed a deep learning framework, Breast Cancer Recurrence Network (BCR-Net), which automatically predicts ODX recurrence risk from histopathology slides. Our proposed framework has two steps. First, it intelligently samples discriminative features from whole-slide histopathology images of breast cancer patients. Then, it automatically weights all features through a multiple instance learning model to predict the recurrence score at the slide level. On a dataset of H&E and Ki67 breast cancer resection whole slides images (WSIs) from 99 anonymized patients, the proposed framework achieved an overall AUC of 0.775 (68.9% and 71.1% accuracies for low and high risk) on H&E WSIs and overall AUC of 0.811 (80.8% and 79.2% accuracies for low and high risk) on Ki67 WSIs of breast cancer patients. Our findings provide strong evidence for automatically risk-stratify patients with a high degree of confidence. Our experiments reveal that the BCR-Net outperforms the state-of-the-art WSI classification models. Moreover, BCR-Net is highly efficient with low computational needs, making it practical to deploy in limited computational settings.

## Background

Breast cancer is the most common cancer in women and the second most common cause of cancer-related death in women [1]. It is estimated that there will be 290,560 new breast cancer cases in 2022, with 43,780 deaths resulting from the disease [1]. The diagnosis, treatment, and prognosis of breast cancer are highly dependent on its classification into distinct histopathological categories [2], stratification into distinct stages [3], grades [4], and receptor status [5]. Receptor status categorizes breast cancer into 3 distinct biological types based on over-expression of estrogen receptors (ER), progesterone receptor (PR), and epidermal growth factor receptor (HER2), namely, hormone receptor breast cancer (positive ER and/or PR over-expression and negative for HER2 over-expression), HER2 positive (HER2 over-expression regardless of ER or PR expression status) and triple negative (negative for ER, PR and HER2). These receptors’ presence or absence is especially important for treatment, as they can be targeted by specific hormonal therapies such as selective estrogen receptor modulators (e.g., tamoxifen), aromatase inhibitors (e.g., anastrozole, letrozole or exemestane) or other biologic agents such as monoclonal antibodies (e.g., trastuzumab or pertuzumab), antibody drug conjugates (e.g., trastuzumab emtansine) or small molecule tyrosine kinase inhibitors (e.g., neratinib) [6, 7].

Approximately 67–80% of breast cancer in women are ER and/or PR-positive (also referred to as hormone receptor or HR positive) [8, 9]. Due to receptor positivity, these cancers can be treated effectively with hormonal therapies such as tamoxifen [10], letrozole [11], and aromatase inhibitors [12] if there is low suspicion of recurrence. Those cancers deemed at high risk of recurrence often require chemotherapy and hormonal therapy [13]. While chemotherapy increases the survival of high-risk patients [14], it has undesirable side effects [15]. Therefore, it is critical to accurately assess the risk of recurrence for ER-positive breast cancer to avoid inappropriately treating patients with chemotherapy which carries a risk of short- and long-term complications.

A common assessment to meet this need is the Oncotype DX (ODX) recurrence score [16–18]. It is a 21-gene assay-based reverse transcriptase-polymerase chain reaction (RT-PCR) quantification that stratifies recurrence risk and predicts benefit from chemotherapy in patients with early-stage hormone receptor-positive, HER2-negative disease based on expression of genes that are involved in proliferation (*Ki67*, *STK15*, *Survivin*, *CCNB1*, *MYBL2*), invasion (*MMP11*, *CTSL2*), ER signaling (*ER*, *PGR*, *BCL2*, *SCUBE2*) (*BRB7*, *erbb2*), and other (*GSTM1*, *CD68*, *BAG1*) with 5 additional “housekeeping” genes serving as reference (*ACTB*, *GAPDH*, *RPLPO*, *GUS and TFRC*) [16]. It yields a recurrence score in a range 0–100, which correlates with the likelihood of breast cancer recurrence after ten years of follow-up and five years of adjuvant endocrine therapy [16]. ODX recurrence score cutoffs also predict the benefit from adjuvant chemotherapy. Reduction in breast cancer recurrence was noted in patients with ODX recurrence score of 16 or greater in women younger than 50 and 26 or greater in women older than 50 with early-stage, node-negative, HR-positive breast cancer who received adjuvant chemotherapy followed by adjuvant endocrine therapy compared to endocrine therapy alone [19]. Conversely, there was no benefit from adjuvant chemotherapy in node-negative patients with ODX recurrence scores below these cut-offs. Similarly, lack of benefit from adjuvant chemotherapy was noted in post-menopausal women and metastases to 1–3 axillary lymph nodes with ODX recurrence score of 25 or less [20]. Unfortunately, ODX and similar gene assays are expensive, time-consuming, and tissue destructive [21–26]. Therefore, many studies seek to predict ODX recurrence risk using more routine and less tissue invasive methods, including MR imaging [27], modified Magee equations [28], nomograms [29], and histopathology [30]. However, these studies are limited in reproducibility since they rely on sophisticated analysis procedures and multiple manually selected variables. To the best of our knowledge, there is still a lack of an end-to-end method to predict ODX recurrence score from medical data.

Predicting ODX recurrence risk from histopathology has garnered particular interest given the ubiquity of routine grading via hematoxylin and eosin (H&E) staining of resection specimens. These methods range from manual [30, 31] to automated [21–24, 32] analysis of digitized H&E images integrated with clinical covariates. Compared to the manual analysis methods, automated methods replace manual feature engineering with automated feature learning, which reduces complexity and human bias in the whole procedure. Up to now, these automated methods relied on the detection of histological primitives (such as nuclei)–sometimes in specific (also automatically detected) anatomical regions (e.g., ducts, tubules, lumen, epithelium, stroma). After detecting these regions and nuclei, features are extracted, and a subset of them are selected based on their discrimination capability. Then, classification is performed into two or three risk categories, often collapsing two categories (intermediate/high) into one. One of the advantages of these automated methods is their interpretability–each extracted feature can be interpreted by humans, such as vascular density or mean tubule/nuclei ratio. However, generating the ground truth for such methods is impractical since they rely on extensively annotated datasets, restricting algorithm development and validation on larger cohorts.

With the recent developments in the deep neural network (DNN) methods [33], especially in weakly supervised DNN [34, 35], whole slide images (WSIs) can be automatically analyzed without the need for exhaustive annotations [36–38]. A DNN consists of multiple learnable "hidden layers", each comprised of a linear function and a non-linear activation function. DNNs can approximate complicated functions while extracting predictive features from data by stacking layers in various combinations. The past decade has seen fully supervised DNN models rise as the most popular paradigm for machine learning. Although promising results have been observed with these approaches in the analysis of small regions of WSIs [39, 40], such DNNs are not feasible without exhaustive and precise tissue-level annotations. In some cases, for example ODX recurrence risk prediction, these tissue-level annotations are not feasible, as the degree to which a local region of tissue contributes to ODX recurrence risk cannot be annotated and labeled by a pathologist. Furthermore, DNNs cannot be applied directly to WSIs as in traditional computer vision application (or patch-wise classification) because fine details such as individual cells, locations, and tissue-level microanatomy (like looking at low-magnification) are lost. On the contrary, weakly supervised DNNs require no human annotation for classification of WSIs [36, 37, 41]. For example, if a tumor comprises a tiny area of a WSI and only the diagnosis (slide-level label) is known, weakly supervised DNNs can learn to correlate implicit tissue-level features (i.e., the tumor) with the slide-level label. This eliminates the need for tissue-level annotations and labels. Furthermore, weakly supervised methods can operate simultaneously at the patch-level and slide-level unlike traditional DNN methods. Given these advantages, weakly supervised approaches are now widely applied to automated analysis of WSIs [37, 42, 43].

Here, we present a weakly supervised method based on attention-based multiple instance learning (MIL) [36], Breast Cancer Recurrence Net (BCR-Net), to predict ODX recurrence risk with minimal manual annotations on H&E and Ki67 images. Our contributions are as follows:

- A novel "intelligent" sampling pre-processing method that leverages slide-level labels to learn a sufficient feature space such that regions predictive of the ODX recurrence risk can be pre-selected from each WSI to minimize downstream training time and improve downstream performance
- A novel application of weakly supervised attention-based MIL to ODX recurrence risk prediction

The proposed method not only outperforms state-of-the-art weakly supervised methods, but it also has the added benefit of being interpretable. Our work also adds to the ever-growing body of evidence advocating using attention-based models to predict clinical outcomes from WSIs [1, 34, 35, 37, 42–47].

## Methods

### Dataset description

This study is IRB approved by the Ohio State University Cancer Institutional Review Board, with a Waiver of Consent Process, and Full of Waiver of HIPAA Research Authorization. Our dataset includes 151 anonymized breast cancer patients. We have access to adjacent pairs of H&E and Ki67 breast cancer resection tissues for 50 patients. We have only access to H&E breast cancer resection tissues for the remaining 101 patients. Ki67 immunohistochemistry was performed using MIB-1 mouse monoclonal antibody from Dako (Santa Clara, CA) on the Leica Bond III system, 1:400 dilution using high pH retrieval (ER2) for 20 min, and the Leica Polymer Refine detection kit. All images were scanned into digital WSIs using a Leica Aperio ScanScope CS2 (Leica Biosystems Inc., Buffalo, Grove, Illinois) at 40× magnification.

**Fig 1:**
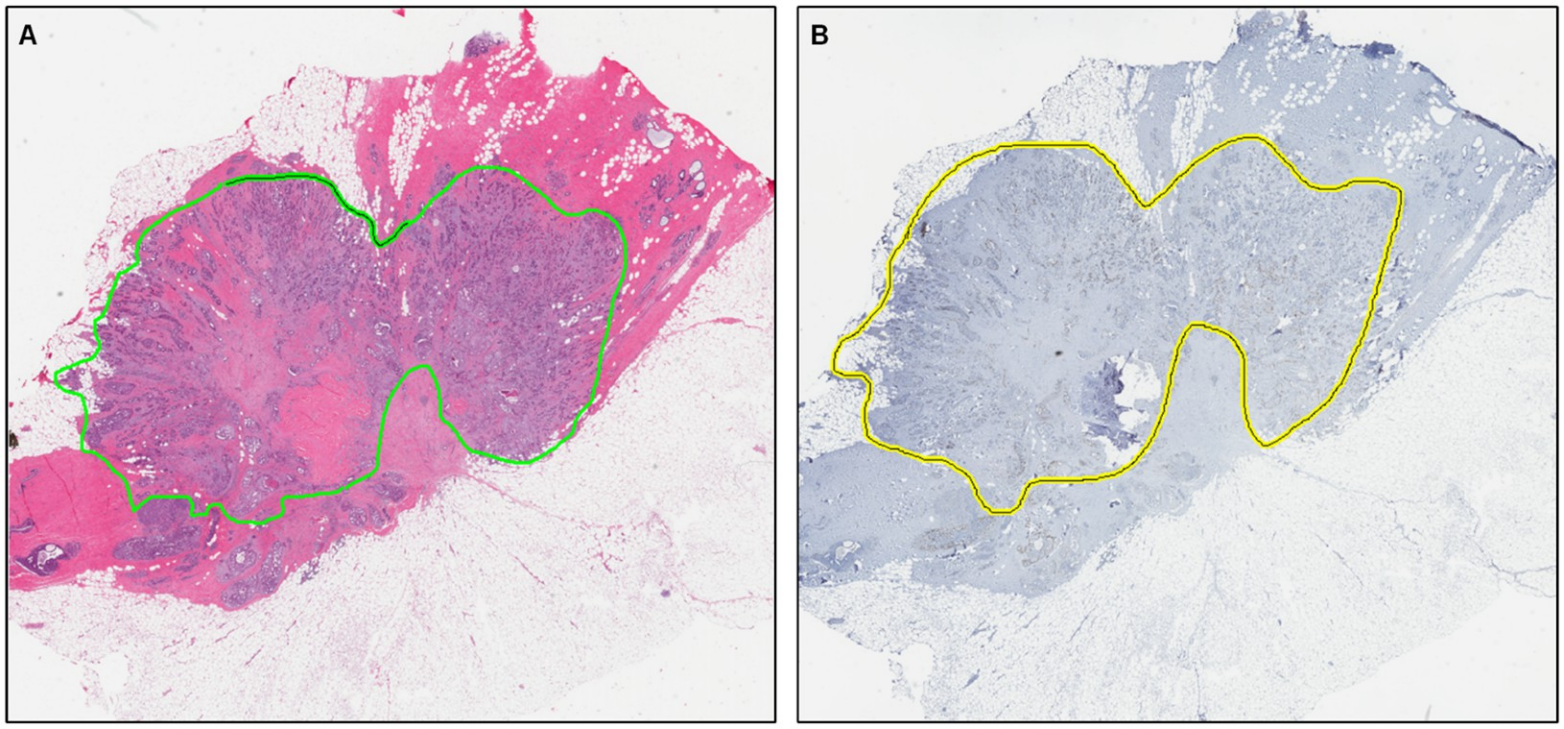
A pair of annotated digital whole slide images. (A) H&E-stained slide. (B) Ki67-stained slide. Tumor regions are annotated in green contour lines for H&E slide and yellow contour lines for Ki67 slide.

Given the most recent research [19], an ODX score of 25 or less is associated with a lack of chemotherapy benefits for women older than 50. On the other hand, for women younger than 50, there is a very modest chemotherapy benefit if the ODX score is 16–25 and no benefit if the ODX score is lower than 16. Thus, we set our ODX score cutoff at 25 –low: if less than 25 and high: if 25 or higher. Our dataset contains WSIs from 64 low-risk and 35 high-risk patients based on this criterion (See Table 1 for dataset information). According to the results of Pearson’s Chi-squared tests, both low and high-risk patient groups have similar distributions in terms of age (p = 0.3681), and histologic type (p = 0.4653). An expert pathologist annotated tumor regions on the slides, which included the region occupied by tumor cells as well as the associated intratumoral and contiguous peritumoral stroma (Fig 1).

**Table 1:** Dataset information—Key characteristics distribution for two risk categories.

Parameters ODX risk categories	Low (< 25)	High (≥ 25)
**Number of patients (N = 151)**	106	45
**Age**		
**0–50**	30	17
**50–80**	76	28
**Grade**		
**1**	47	2
**2**	48	20
**3**	11	23
**Histologic type**		
IDC*	91	41
**Lobular**	12	3
**Tubular**	1	0
**Micropapillary**	0	1
**Mucinous**	2	0
**Receptor status**		
**HR+ and HER-**	106	45

* IDC: Invasive ductal carcinoma

### Methodology

The framework consists of two steps: (1) intelligently sample patches with a pretrained patch-level CNN-scorer and (2) classify slides with MIL model.

#### Justification for intelligent patch sampling

**Fig 2:**
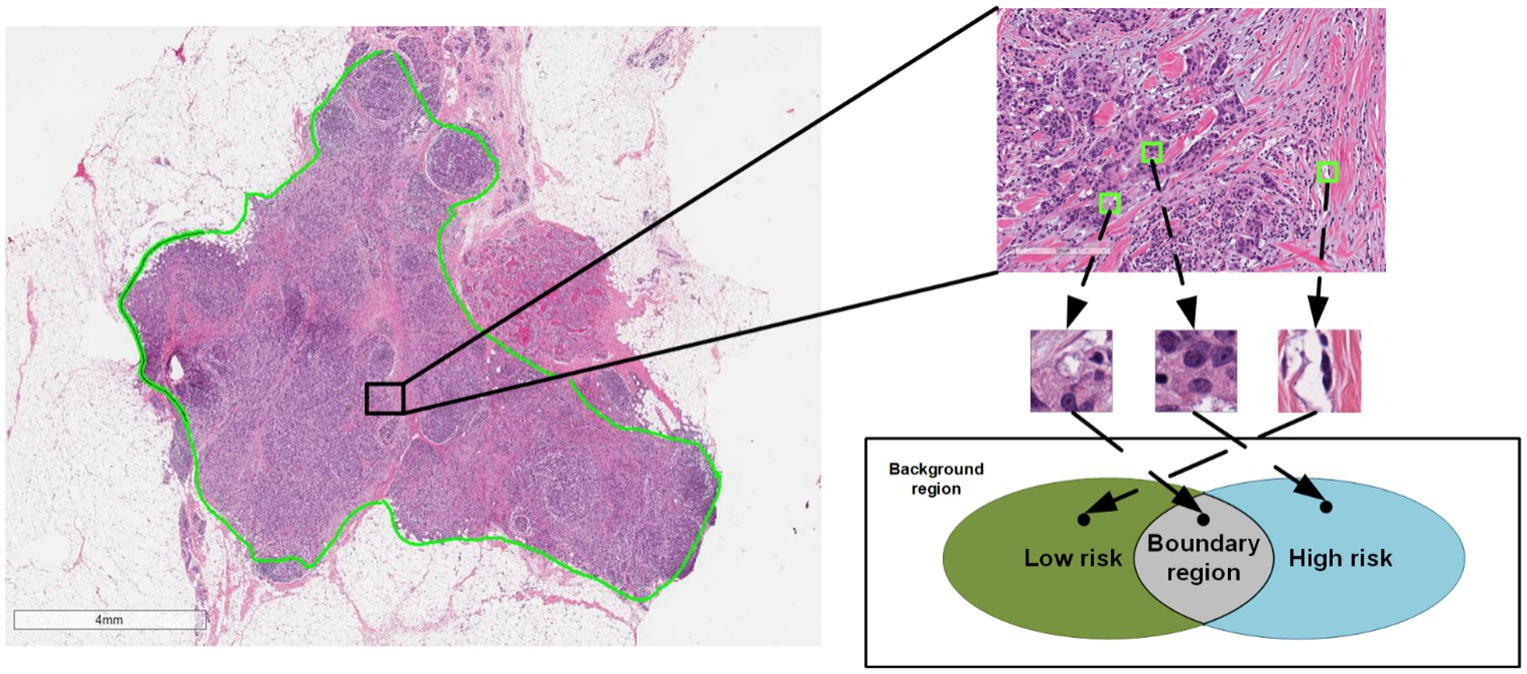
Illustration of the three categories for intelligent sampling. The image on the left side is a sample slide of our dataset where the tumor region is annotated in green line. Three example patches from an example region (shown as a black rectangle and magnified on the right side) are cropped from this example region (shown in green squares) and belong to different sections of the Venn diagram in the bottom. This Venn diagram demonstrated different implicit categories of patches of the WSI. The discriminative patches in green and blue section of the diagram are patches we want to sample for better slide-level classification.

Generally, when a WSI is divided into small image patches, each patch belongs to one of three categories (Fig 2):

1. Discriminative patches: those only correlated to one slide-level label (i.e., outcome), e.g., low risk, or high risk
2. Non-discriminative patches: those that are moderately correlated to multiple slide-level labels (i.e., contained heterogeneous information and are hard to be classified), and
3. Background patches: those that are uncorrelated to any slide-level label.

We illustrate the three categories in the Venn diagram (See Fig 2). Inside the tumor region, the patches that are only correlated to one clinical output are the **discriminative patches**. As shown in the Venn diagram, the green region is the set of patches from low-risk category, and the blue region is the set of patches from high-risk category. Discriminative patches from different categories will clearly differ for the model to learn the decision boundary and separate them. While inside the tumor region, some patches do not contribute any meaningful information to machine learning models and contribute to noise. These patches constitute the **non-discriminative patches** set, which is the grey region in the overlapping region of the Venn diagram. In our recurrence risk prediction problem, the patches outside the tumor region constitute the **background patches** set because they are uncorrelated to any clinical outcome. With the tumor region annotated, the background patches in our dataset were removed and not considered in the proposed method. An innovative contribution of BCR-Net is to automatically select discriminative instances (i.e., patches) from WSIs using CNN-scorer.

#### Intelligent sampling in patch-level

**Fig 3:**
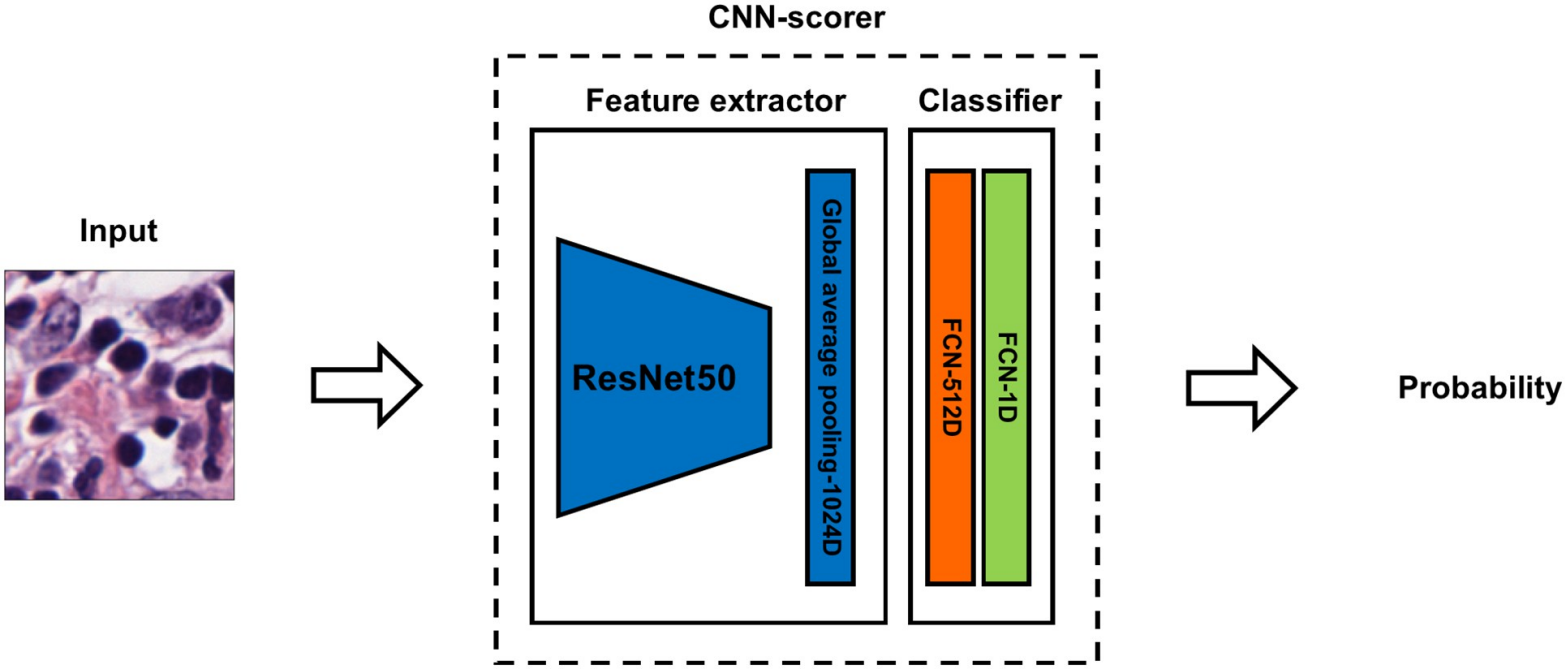
The CNN-scorer architecture. The input size of the model is 224×224. The feature extractor will map the input patch to a 1024-dimension feature vector. Then, the feature vector will be further mapped to a scalar which will be used to compute the discriminative score (See Eq 1).

To intelligently sample those discriminative patches, we propose CNN-scorer, a convolutional neural network (CNN), to score patches from the WSIs (Fig 2 depicts this concept as a Venn diagram). The purpose of the CNN-scorer is to score patches based on their ability to predict slide-level labels. The overall architecture of our CNN-scorer is shown in Fig 3. It is an ImageNet pre-trained ResNet50 (truncated from the third residual block) [48, 49] followed by a global pooling layer, two fully connected layers, and sigmoid activation as output. The sigmoid activation function is widely used to predict an input patch’s probability of "membership" to either class in the binary classification problem. The output ranges from 0 to 1, and a threshold of 0.5 is applied to distinguish between classes. Patches with a probability close to the boundary values (i.e., either 0 or 1) should indicate a high confidence class 0 or class 1, respectively, indicating that those patches are discriminative for their own classes. On the other hand, probabilities near 0.5 should be non-discriminative. Using these assumptions, we can derive the discrimination score (DS) of a patch by subtracting 0.5 from its probability and taking the absolute value in this form:

$$DS=\frac{\left|\sigma \left(f_{\theta }\left(x\right)\right)-0.5\right|}{0.5},$$ (1)

where *f*_*θ*_(·) is the CNN model, *θ* represents the parameters inside the CNN model, *σ*(·) is the sigmoid activation function, and x is the input image.

The DS values range from 0 to 1, with a higher DS indicating that the patch has greater discriminative capacity. As a result, we sample patches with high DSs for slide-level classification. For the training of this CNN model, we build a patch-level training set which is a randomly sampled set of cropped patches. Although patch-level labels are not available, a weakly supervised strategy can be utilized in which patch-level labels take on their slide-level label during training. The CNN learns to map each patch to its label (i.e., low/high risk). Once the CNN model is trained, Eq 1 is applied to the output of the CNN to compute DSs. For training details, please see Section 2.3.1.

**Fig 4:**
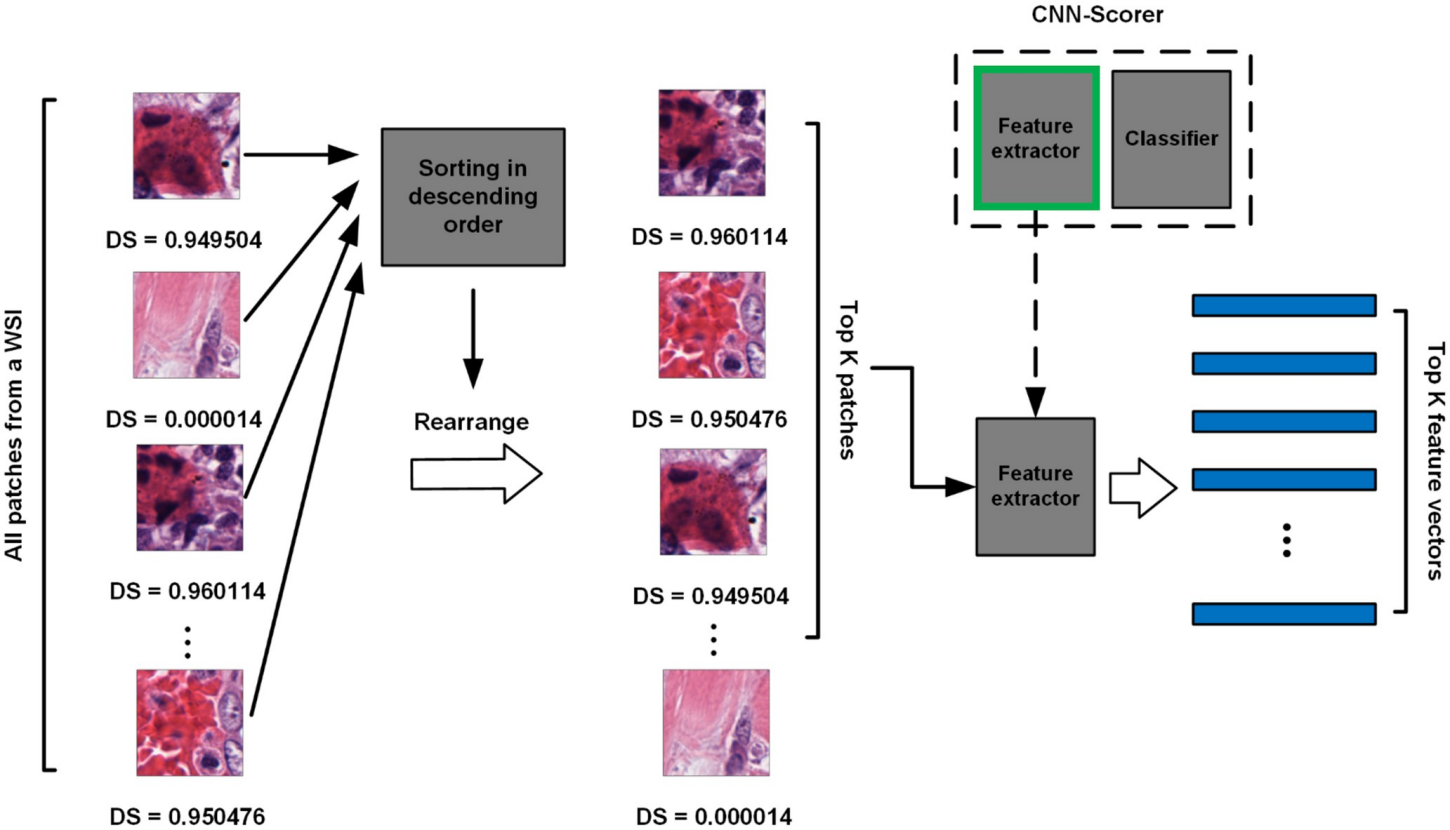
Intelligent sampling. All the patches from the tumor region of a WSI are scored by the pretrained CNN-scorer and then rearranged according to their DSs in descending order. The top K patches are sampled as the most discriminative for this WSI. The sampled patches are fed into the same CNN as a feature extractor (i.e., without the classification head) to be embedded into feature vectors. The top K extracted feature vectors act as a bag of instances for subsequent training of an MIL model.

Using this pretrained CNN-scorer, all patches from the tumor region of each WSI can be scored. Then, patches are sorted from high to low according to their DS. The top K patches are then sampled as the most discriminative patches of the WSI for slide-level classification. This process is shown in Fig 4. We select high-ranking patches instead of setting a constant threshold value because the scores of patches from different slides will be in different distributions. Thus, a constant threshold value will not be effective for sampling all slides. All sampled patches are then fed into the same CNN-scorer without a classification layer (i.e., after flattening) to be embedded into feature vectors. With the input patch size of 224 × 224 in our experiments, the embedded feature vectors are in 1024 dimensions. As a result, this process yields a set of K feature vectors as a bag for each WSI which will be used for subsequent training of an MIL model for slide-level classification. The overall intelligent sampling process is shown in Fig 4.

#### Multiple instance learning for slide-level classification

Given a bag of sampled feature vectors, we formulate the slide-level classification problem into a MIL problem. MIL is a machine learning paradigm in which labels are assigned to collections of data points ("bags") rather than individual data points ("instances") in some datasets. In this manner, each of the selected and extracted feature vector from a WSI is an instance, and the collection of those feature vectors will be a bag that represents this WSI. The classification in MIL is done at the bag level, which is slide-level in our problem.

MIL is conventionally posed as a two-class problem, where bags are either assigned a "positive" or "negative" label. Similarly, instances of each bag have a positive or negative label. The three main underlying assumptions of MIL relate to bags and their instances. First, instance labels are not explicitly assigned or known; they implicitly exist. Second, positive bags must contain positive instances and may contain negative instances. Third, negative bags must only contain negative instances [38].

A useful analogy to understand the MIL paradigm is a disease on the tissue level. Here, an instance can be thought of as a tissue region, and a bag can be thought of as a collection of tissue regions from an individual. Each region of the tissue (i.e., bag) is either diseased (i.e., positive) or healthy (i.e., negative). This is determined by their tissue, which, when examined one region at a time (instance), will similarly present as diseased (positive) or healthy (negative). Tissue from a diseased individual (positive bag) will contain diseased (positive instances) tissue and may contain healthy (negative instances) tissue. In contrast, tissue from a healthy individual (negative bag) will contain only healthy tissue (negative instances).

**Fig 5:**
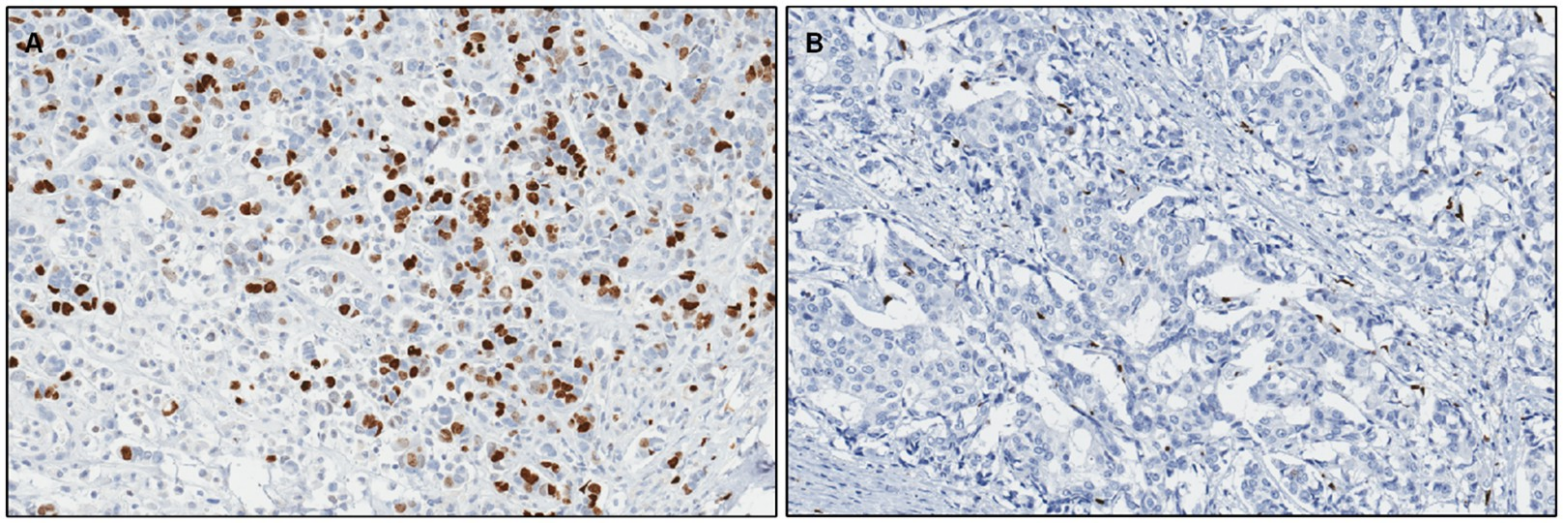
The high ODX risk slides usually have a higher density of PCs (stained in brown) compared to the low score slides, which can be clearly demonstrated in Ki67-stained slides. (a) Example tiled region from a high-risk slide. (b) Example tiled region from a low-risk slide.

We observe that patients with high ODX risk have a higher density of proliferating cells (PCs) compared to patients with low ODX risk (Fig 5). In the MIL context, we can formulate the low-risk slides as negative data, which only contains patches with sparse PCs, while the high-risk slides as positive data, which contains both sparse and dense patches of PCs.

Instance pooling is the core component of MIL models, which specifies how instances are combined into a single, "bag-level" representation. For example, in examining multiple tissue regions on a slide, a pathologist must combine (i.e., pool) the individual (instance) analyses to reach the final decision. Typically, pooling fuses abstract representations of instances (i.e., feature vectors in our case) into a single meta-instance. This meta-instance is then further processed (i.e., some prediction is made with it). Common pooling methods include max pooling, mean pooling, and log-sum-exp (LSE)-pooling [50], and attention-based pooling [36].

**Fig 6:**
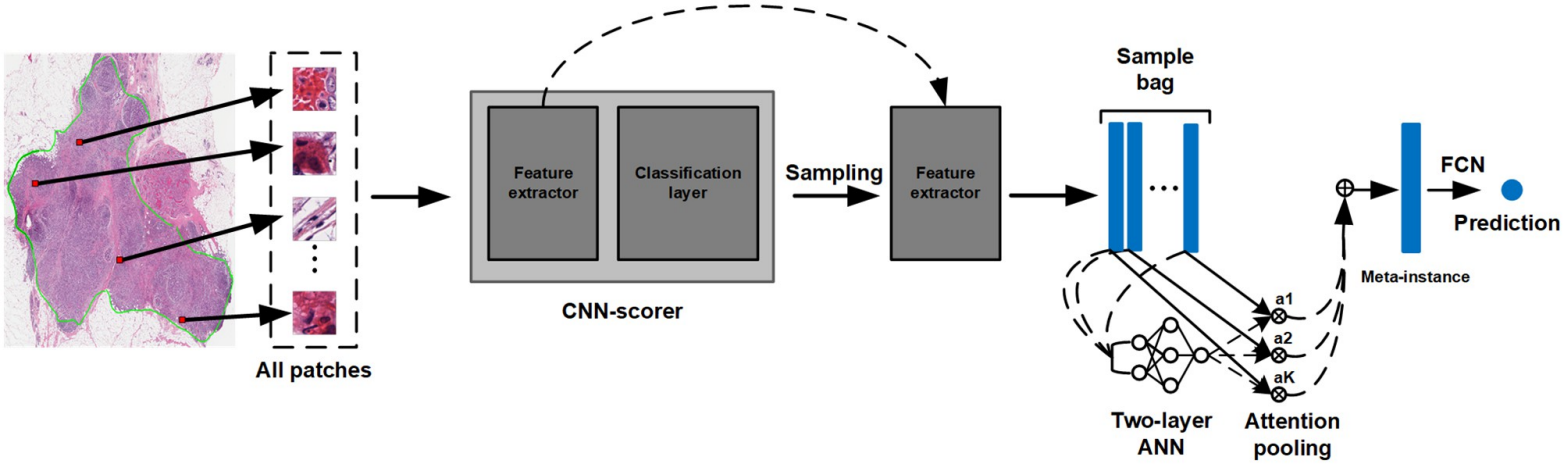
The proposed BCR-Net model. All patches from the WSI’s tumor region (annotated in green) are fed into a pretrained CNN-scorer. The patches are rearranged according to their DSs as previously defined (see Eq 1) in descending order. The top K patches are sampled and embedded by the same feature extractor inherited from the CNN-scorer (Fig 4). The output K feature vectors are treated as a bag of instances and aggregated through attention-based pooling. As per the attention-based pooling, the attention weights a1, a2, …, aK are produced by the ANN. And then, a weighted sum is conducted to aggregate the feature vectors with their attention weights. As the final representation of the WSI, the output meta-instance is classified by a fully connected layer (FCN) and a probability score will indicate the final pre-diction for the WSI.

In our BCR-Net, we implement attention-based pooling [36]. It is unique in that it automatically learns a function to combine multiple instances into a single meta-instance rather than hard coding a function (e.g., average, max, or summation). Our implementation consists of a learnable two-layer artificial neural network (ANN) that maps instance, from an intelligently sampled bag in Section 2.2.2, to a single value, aptly called an attention weight (see Fig 6). Namely, in one training step, all instances of a bag will be fed into the ANN in parallel. Then, the meta-instance is computed using a weighted sum of each instance and its respective attention weight. The mathematical definition of the attention pooling is:

$$M\left(x_{k|k=1\ldots K}\right)=\sum_{k=1}^{K}a_{k}x_{k},$$ (2)

where:

$$a_{k}=\frac{exp\{w^{T}\left(tanh\left(Vx_{k}^{T}\right)⊙sigm\left(Ux_{k}^{T}\right)\right)}{\sum_{j=1}^{K}exp\left\{w^{T}\left(tanh\left(Vx_{j}^{T}\right)⊙sigm\left(Ux_{j}^{T}\right)\right)\right\}},$$ (3)

where $x_{k|k=1\ldots K}\in {\mathbb{R}}^{1\times 1024}$ are the instance embeddings inside the bag, *K* is the number of instances in the bag, $a_{k}\in {\mathbb{R}}^{1\times 1}$ is the attention weights of ***x***_*k*_ learnt by the ANN and *M*(***x***_*k*|*k* = 1…*K*_) is the meta-instance of the bag. As shown in Eq 3, $V\in {\mathbb{R}}^{512\times 1024}$ and $U\in {\mathbb{R}}^{512\times 1024}$ composed the parameters of the first layer of the ANN, and ⊙ means element-wise product. We applied weight normalization to **V** and **U** layers to stabilize the optimization during the training [51, 52]. Their outputs are activated by tanh and sigmoid activation functions, and then the element-wise product is applied to the two outputs. The $w\in {\mathbb{R}}^{512\times 1}$ is the parameter of the second layer of the ANN. The outputs of the second layer are then normalized by the normalized exponential function (i.e., softmax function). Then, in Eq 2, the yielded attention weights *a*_*k*_ will be used to aggregate the instances ***x***_*k*_.

The magnitude of an attention weight correlates with how important its respective instance is in the downstream prediction on the meta-instance. It is important to use attention weights to further highlight some discriminative instances, since the selected bag contain discriminative instances for both low- and high-risk categories (See Section 2.2.2, Eq 1). As a result, the attention weights may reveal which regions of a slide correlate with that slide’s overall label. Finally, the meta-instance is classified by a fully connected layer (FCN) and a probability score will indicate the final prediction for the WSI. The loss of the final prediction for the meta-instance will be backpropagated to the ANN, so that the ANN will learn to assign reasonable “attention” to each instance according to its importance.

#### Comparison with other MIL methods

We compared the performance metrics of our method to the state-of-the-arts MIL models for the WSI classification, which are CLAM and TransMIL [37, 53]. CLAM also utilizes attention [36] to dynamically learn and fuse features predictive of the desired outcome (in our case, ODX recurrence risk). TransMIL employs the vision transformer architecture [54] that enables the MIL model to capture both the morphological and spatial information of the WSIs. Both methods are highly robust and well-known for their ability to generalize to multiple WSI datasets. We used authors’ recommended hyperparameters when reproducing CLAM and TransMIL in order to demonstrate their best performance. The remaining components of the experimental design were identical when comparing these two methods with the proposed BCR-Net.

### Experimental design

Fig 6 depicts our overall proposed methodology. We utilized ODX recurrence risk as slide-level labels (high vs. low) for our classification task. For H&E-stained slides, we conduct 5-fold cross-validation and hold-out testing. We randomly split the data into training (n = 72), validation (n = 8), and testing (n = 18) datasets, in which the training and validation sets are randomly split for five times and testing set is hold-out for all the folds. We balanced the number of low and high-risk data by sampling equal number of patients from low-risk cohort. In each fold, the validation set was used for model training and parameter optimization and the testing set is used to test the model’s performance.

For Ki67-stained slides, we conducted leave-two-out-cross-validation (LTOCV), in which one slide from each class was taken for each validation set. As a result, there are a total of 25 folds, each with 48 WSIs as the training set and two WSIs as the validation set. The training set was used for model training and parameter optimization in each fold, while the validation set was used to test the model’s performance. Our code and some example data are publicly available on https://github.com/JoeSu666/BCRNet.

#### Training of CNN-scorer

In each fold’s experiment, we built a patch-level training set specially for the CNN-scorer. Namely, we randomly selected 200 patches from each WSI in the current fold’s training WSIs, resulting in a total of 14000 patches. Each patch was labeled with its slide-level label. We used the binary cross-entropy loss function for training these models. The model was optimized using Adam with a learning rate η of 0.0002 for a maximum of 150 epochs. To avoid overfitting, we saved the CNN-scorer when the training accuracy didn’t improve for 15 epochs.

#### Training and validation of attention-based MIL model

For each fold, with the CNN-scorer pre-trained, we conducted intelligent sampling on patches from both training and validation WSIs. To examine the influence of K’s value, we conducted experiments with the top 1500-, 3000-, 5000-, 8000-, and 10000-sample bags. The resulting bags of feature vectors were used for training and validation of the attention-based MIL model. Binary cross-entropy was used as a loss function. The model was optimized using Adam with a learning rate η of 0.0002 for a maximum of 150 epochs. An early stopping strategy was applied to avoid overfitting when the validation accuracy (training accuracy for Ki67 experiments) did not improve for 15 epochs. Experiments were carried out only on the annotated tumor region of H&E and Ki67-stained slides with 224×224 patch size at 40x magnification.

## Results

### Intelligent sampling with CNN-scorer

**Fig 7:**
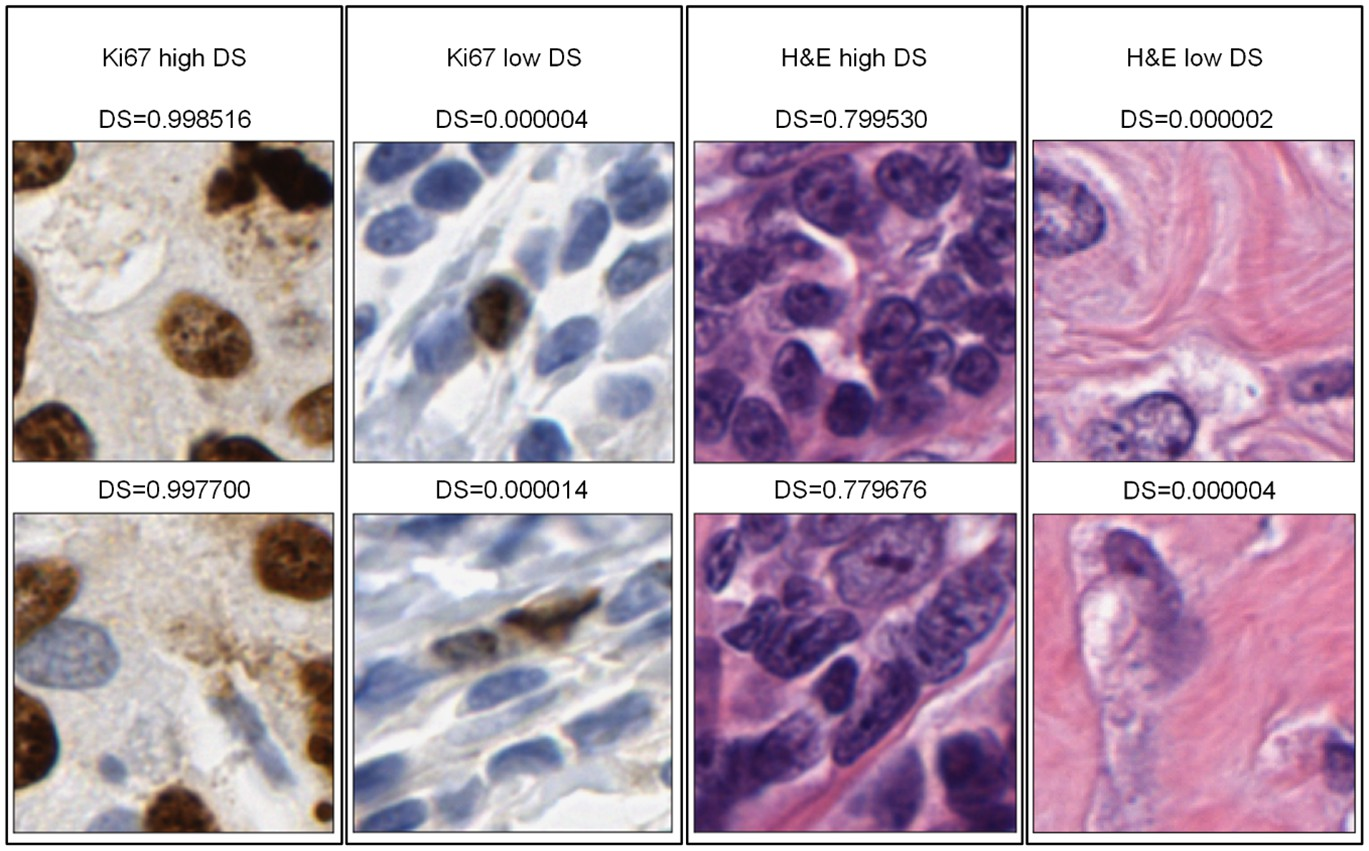
Example scoring results of Ki67 and H&E patches. Examples were selected among patches who received top 50 high DSs and bottom 50 low DSs in their slides from the CNN-scorer. DSs are ranged from 0 to 1. The higher the DS is, the more discriminative the DS is.

Fig 7 depicts some sample outputs for selected patches. There are clear similarities between the high DS images as well as those with low DSs both in H&E and Ki67-stained images.

### Predicting ODX recurrence risk

The slide-level ODX prediction results of BCR-Net and comparison method [37] on H&E-stained slides are shown in Table 2. Here, models are evaluated on the hold-out testing set in the 5-fold cross-validation. Then, values reported are reported with mean ± standard deviation across the five folds.

**Table 2:** Slide-level hold-out testing results on H&E-stained slides. K = 5000 for BCR-Net results. Values are reported with mean ± standard deviation across the five folds.

Method	AUC	Accuracy	Low risk	High risk	F1-score
**CLAM [37]**	0.716±0.022	0.644±0.027	0.688±0.147	0.600±0.166	0.617±0.070
**TransMIL [53]**	0.6321±0.054	0.556±0.049	0.578±0.237	0.533±0.285	0.546±0.091
**BCR-Net**	**0.775±0.079**	**0.700±0.030**	**0.689±0.049**	**0.711±0.099**	**0.700±0.051**

The slide-level ODX prediction results of BCR-Net and comparison method [37] on Ki67-stained slides are shown in Table 3. Here, models are evaluated on the validation sets during the LTOCV. Then, values are reported with mean and 95% confidence interval (CI) across all the folds. The CIs were computed using the bootstrapping method.

**Table 3:** Slide-level validation results of LTOCV on Ki67-stained slides. K = 3000 for BCR-Net results. Values are reported with mean and 95% CI (in []).

Method	AUC	Accuracy	Low risk	High risk	F1-score
**CLAM [37]**	0.713 [0.685, 0.732]	0.720 [0.704, 0.741]	0.808 [0.778, 0.821]	0.625 [0.612, 0.672]	0.681 [0.650, 0.673]
**TranMIL [53]**	0.790 [0.773, 0.801]	0.712 [0.699, 0.723]	0.704 [0.690, 0.724]	0.720 [0.706, 0.724]	0.706 [0.687, 0.708]
**BCR-Net**	**0.811 [0.793, 0.819]**	**0.800 [0.789, 0.808]**	**0.808 [0.788, 0.815]**	**0.792 [0.776, 0.808]**	**0.792 [0.766, 0.793]**

**Fig 8:**
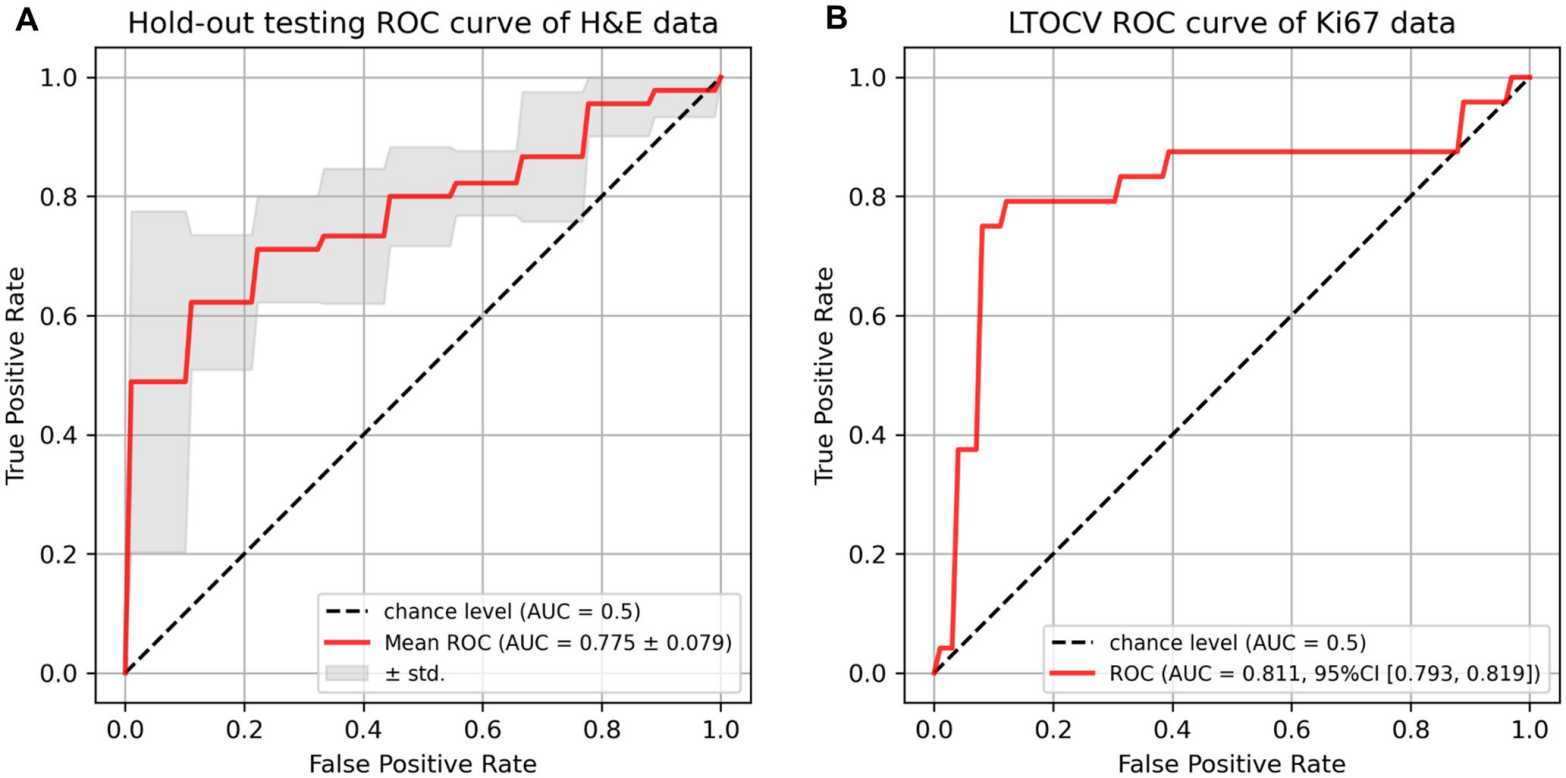
Receiver operating characteristic (ROC) curves of BCR-Net on H&E and Ki67-stained slides. (a) Hold-out testing ROC curves on H&E-stained slides. The red curve indicates the mean curve based on the five folds’ testing results and the gray shadow indicates the standard deviation. (b) LTOCV ROC curves on the Ki67-stained slides. The red curve indicates the mean curve based on the validation results of the LTOCV.

The Receiver operating characteristic (ROC) curves of BCR-Net on H&E and Ki67-stained slides are shown in Fig 8.

### Ablation study

**Fig 9:**
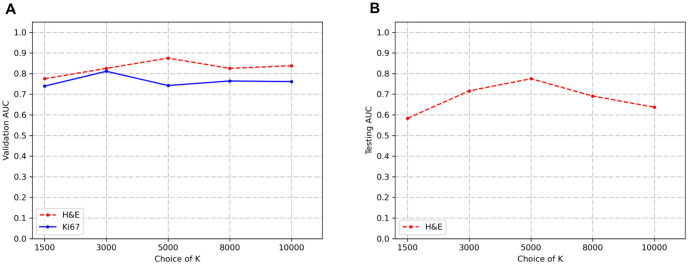
Validation and testing AUC for slide-level classification in low and high-risk ODX using BCR-Net with different sample size K. (a) Mean cross-validation AUC of 5-fold cross-validation for H&E-stained slides and LTOCV for Ki67-stained slides. The best validation AUC for H&E-stained slides is 0.875 with K = 5000. The best validation AUC for Ki67 stained slides is 0.811 with K = 3000. (b) Mean hold-out testing AUC of 5-fold cross-validation for H&E-stained slides. The best testing AUC for H&E-stained slides is 0.775 with K = 5000.

In order to show the influence of K to the BCR-Net performance, we conducted an ablation study on the choice of K. Fig 9 depicts the prediction AUC when different numbers of K top patches are used to construct the bag as the input of the subsequent MIL model. From the figure, we find that the validation AUCs increase as the number of samples increases. However, AUCs reach a steady-state value when the number of samples in the bag exceeds a certain number. The best prediction performance for H&E and Ki67 stained slides yielded by K = 5000 and K = 3000 models correspondingly. Additionally, we investigated the patch size’s influence on our model and exhibited the results in Table 4.

**Table 4:** Slide-level hold-out testing AUCs on H&E-stained slides using BCR-Net (K = 5000) in different patch sizes. Values are reported with mean ± standard deviation across the five folds.

Patch size	112×112	224×224	448×448
**AUC**	0.649±0.022	0.775±0.079	0.751±0.068

**Fig 10:**
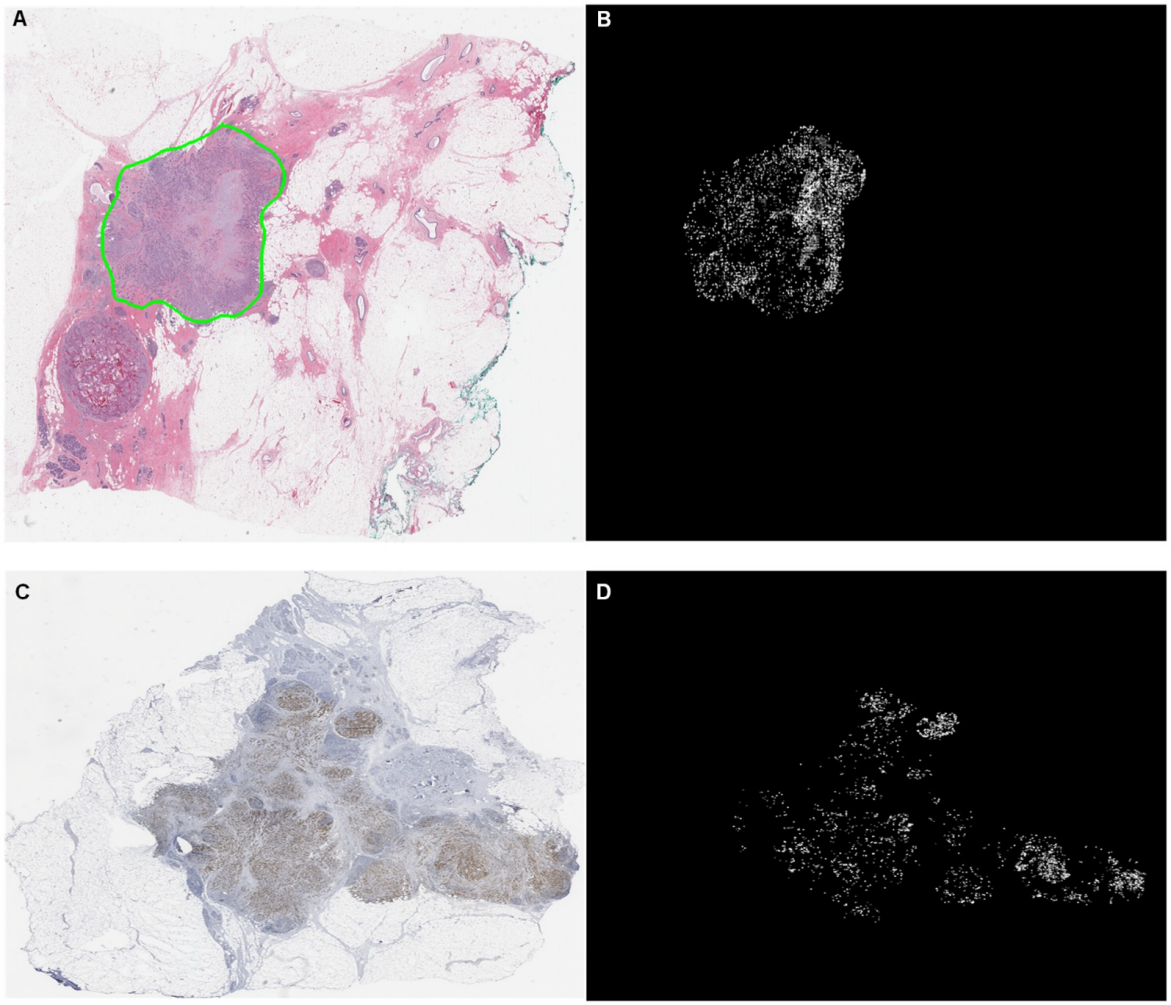
Heatmap visualization of the attention weights yielded by the attention based MIL model. The top row are a thumbnail and corresponding heatmap of an H&E slide. The bottom row are a thumbnail and corresponding heatmap of a Ki67 slide. The heatmaps are contrast enhanced for visualization purpose. The bright area in the heatmaps correspond to WSI area that receive high attention weights.

**Fig 11:**
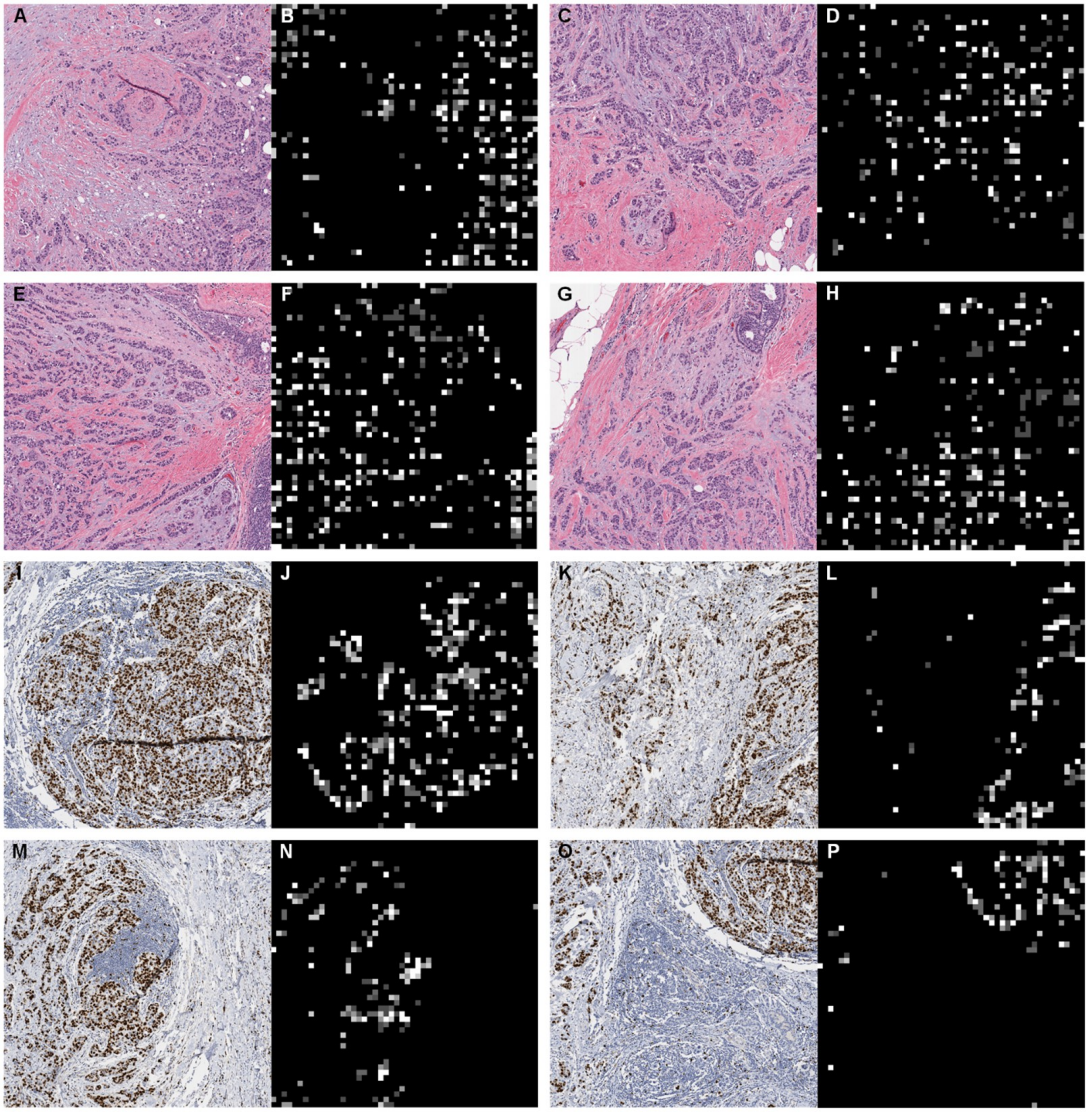
Magnified local regions and corresponding heatmaps. We can clearly find that the attention based MIL is highlighting specific tissue patterns. This is especially interpretable on Ki67 images, where proliferating cells (i.e. brown color regions on WSI images) are assigned with high attention weights (i.e. bright regions on heatmaps).

In order to demonstrate the interpretability of the BCR-Net, we visualized the attention weights that were assigned to the WSI regions by the attention module of BCR-Net. We visualized the attention in the form of a heatmap, where each patch on the WSI was assigned the value of its attention weight (see Figs 10 and 11). We can easily observe that BCR-Net is paying attention to specific tissue patterns from Figs 10 and 11 From Fig 11I–11P, we find that proliferating cells (i.e. brown color regions on WSI images) are assigned with high attention weights (i.e. bright regions on heatmaps).

**Fig 12:**
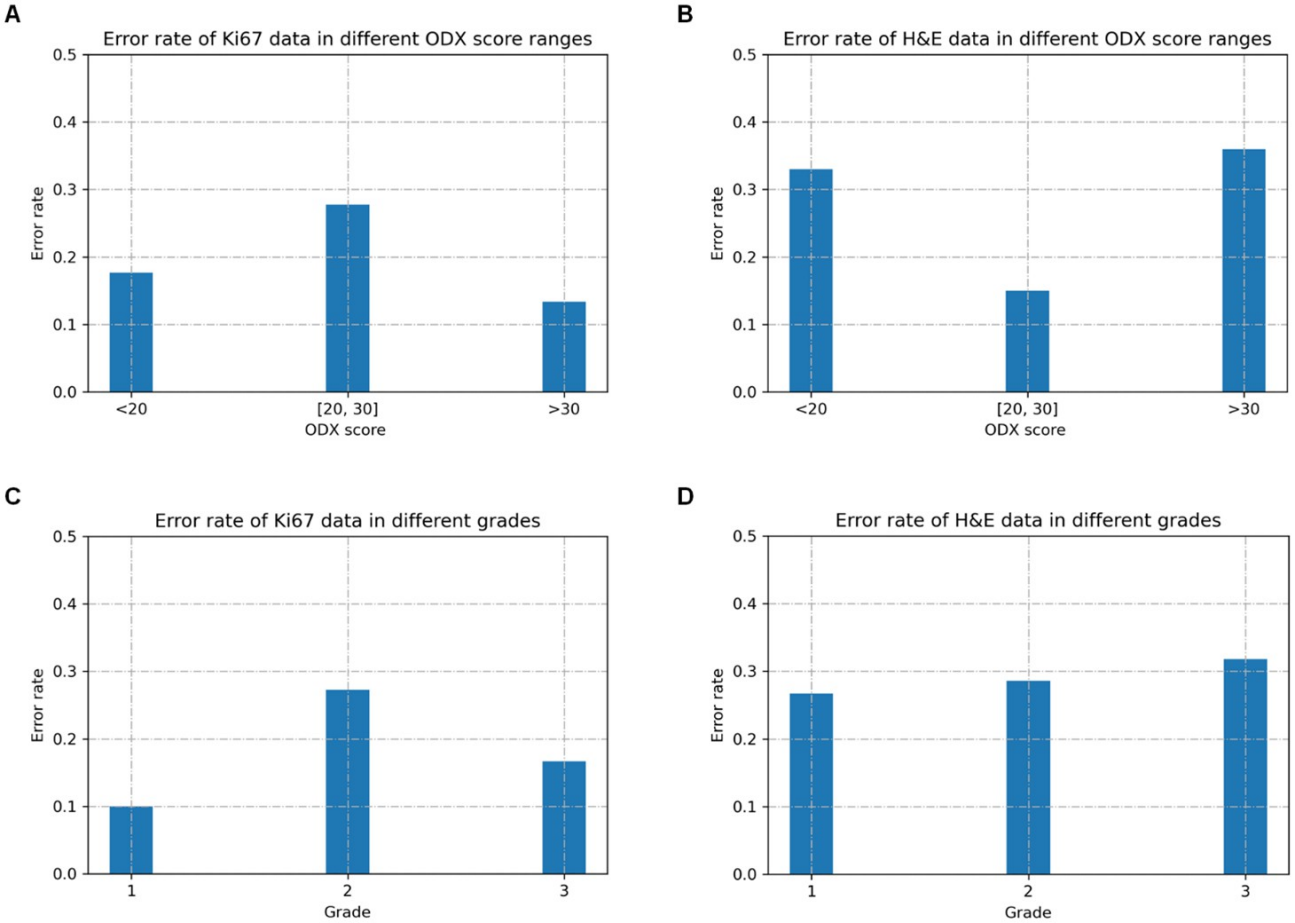
BCR-Net’s validation error rate for patients in different ODX score ranges and grades. The error rate in a particular group is calculated by dividing the number of misclassified cases in that group by the total number of cases in that group during cross-validation. (a) Error rate resulting from using Ki67 data in different ODX score ranges. (b) Error rate resulting from using H&E data in different ODX score ranges. (c) Error rate resulting from using Ki67 data in different grades. (d) Error rate resulting from using H&E data in different grades.

We also analyzed the BCR-Net’s validation error rate for patients in different ODX score ranges and grades (See Fig 12). We can conclude how different oncological factors correlate with (i.e., borderline ODX scores, certain grade types) our prediction.

**Fig 13:**
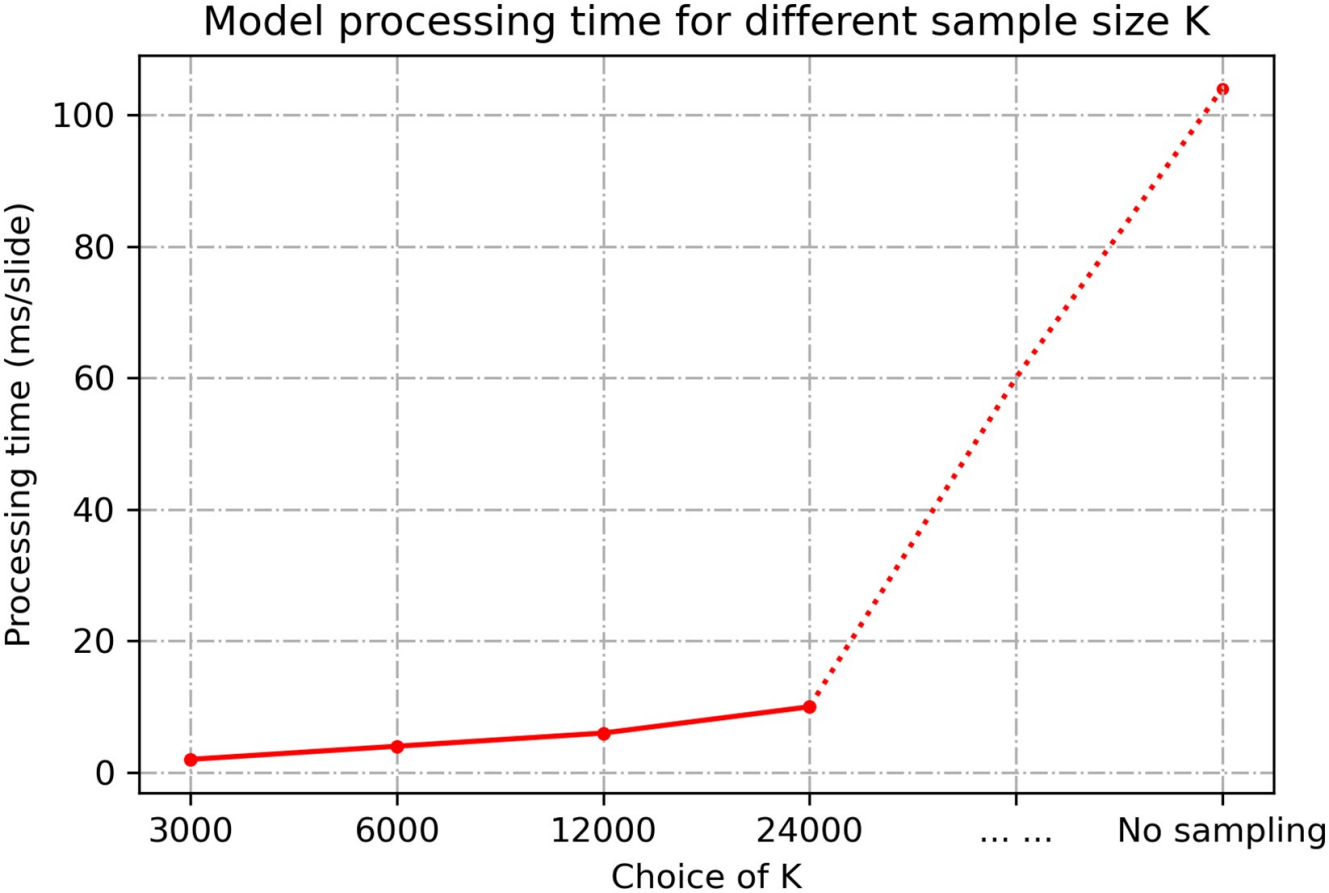
Model processing time for different sample size K. We evaluated the time of making prediction for one slide using the proposed BCR-Net under different sample size K. The red dot indicated the processing time for different choices of K. No sampling means that the intelligent sampling was not applied (i.e., all feature vectors were in the bag).

To demonstrate our sampling strategy’s computational efficiency, we also compared the proposed model’s computational speed to make the prediction for one slide when using different sample sizes and using all samples (i.e., no sampling). The results, shown in Fig 13, indicate that it takes, on average, 2–4 ms to process a WSI for K values changing between 3000 and 24000. When there is no sampling, it takes 104 ms to process a single WSI. The proposed intelligent sampling makes the prediction 50 times faster than the method without sampling. Our experiments were implemented on a workstation with one NVIDIA P100 GPU.

## Discussion

This study presents a novel deep learning-based method, BCR-Net, to predict the ODX recurrence risk without exhaustive patch-level annotations automatically. The novel contributions of this method are: 1) an intelligent sampling method that can efficiently select discriminative local regions (i.e., patches) from the WSIs without nuclei level tumor annotation; 2) a multiple instance learning classifier that can accurately predict the ODX recurrence risk of gigapixel WSIs with low computational cost in terms of speed.

Our primary objective for the proposed method is to automatically predict the breast cancer recurrence risk compared to the ODX recurrence risk. Compared to the manual method [27–31], automated methods can give more accurate outcomes with low human bias [21–24, 32]. New deep learning methodologies produce especially promising prediction accuracies, taking advantage of the advanced computational resources and large data availability. However, most of those methodologies require tissue-level annotation on WSIs, a major limitation for using them to train and test deep learning algorithms. In most cases, each WSI only has a slide-level annotation corresponding to some tiny regions compared to the gigapixel-level image. This kind of limited data results in insufficient training for ordinary deep learning models, resulting in low accuracies or generalization issues. In addition, the sheer size of the WSI can overwhelm ordinary computational resources during deep learning training.

We presented a weakly supervised intelligent sampling method to automatically select the discriminative patches from a WSI to overcome these challenges. Trained with slide-level labels, our patch-level CNN-scorer can select the most discriminative patches closely related to the ODX recurrence risk. The proposed method is fast because it is trained on sampled patches from each slide instead of the whole slide. Additionally, the proposed method utilizes an attention-based multiple instance learning [36] on the sampled bag of patches to produce the slide-level prediction. This method treats each patch as an instance inside the sample bag. The attention-based MIL uses a self-supervised strategy to weigh each instance according to its significance to the slide-level prediction. This approach further highlights the discriminative patches from the roughly selected sample bag by CNN-scorer. As a result, we can obtain a more accurate prediction of the ODX recurrence risk.

We are not the first to apply attention-based MIL on pathological whole slide images. Lu et al. recently proposed an attention-based CLAM model for multi-class WSI classification tasks, reaching the state-of-the-art accuracy [37]. However, their method uses features derived from ImageNet, mainly consisting of common, everyday objects that bear no resemblance to cells and tissues’ morphological characteristics. Thus, models trained on features yielded from ImageNet do not create a feature space to discriminate WSI patches accurately. On the other hand, in the BCR-Net method, the features are weak but relevant to histopathology. Using the same feature extraction model for both the patch-level and slide-level classifier, the boundaries between low-dimensional patch representations are more easily learned and refined by the slide-level MIL classifier.

Based on the experimental results, we noticed that the proposed method produces promising results, outperforming the comparison methods (CLAM and TransMIL) on both H&E and Ki67 stained slides. The results show that the CLAM model performed well on Ki67 stained slides but overfits to one of the classes on H&E stained dataset. Furthermore, the TransMIL model achieved relatively low accuracy on both the H&E and Ki67 datasets, which could be attributed to the fact that the vision transformer-based models require large dataset for training [54]. The results show that the proposed model performs better on both, Ki67 and H&E datasets. This could be explained by the fact that Ki67 staining reflects tumor proliferation, which is directly correlated to the prognosis [55]. Even so, our performance on H&E slides is significant. As the H&E-stained slides are readily available at most hospitals, our method can be developed as a web-based system and can be accessible in many parts of the world with an internet connection at a much lower cost than ODX.

The results from Fig 9 indicate that the model achieved the best performance for a certain sampling size, and then the performance degraded with more samples included in the bag. This finding is consistent with the intended purpose of conducting intelligent sampling, which is to remove ambiguous patches and thus improve the slide-level prediction accuracy. The results from Table 4 show that our model achieves outstanding AUC in 224×224 and 448×448 patch sizes while achieving modest AUC in 112×112 patch size. A possible explanation for this might be that the field of view of 112×112 patch under 40× magnification is too small for the DNN model to correlate image features with the diagnostic information. Our model also exhibits great visual interpretability. From Figs 10 and 11, we clearly found that the attention module of BCR-Net is paying attention to specific anatomical patterns of H&E and Ki67 stained tissues. According to the heatmap, the proliferating cells (i.e. stained in brown) are highlighted by the attention weights (see Fig 11I–11P). Proliferating cells were found to be related to a high ODX risk [16]. Although imaging biomarkers of ODX risk on H&E-stained slides are not easily interpretable, pathologists can utilize our BCR-Net to explore new biomarkers from WSIs with the strong interpretability of BCR-Net. Moreover, we investigated different ODX score ranges and grades’ influence on BCR-Net’s prediction accuracy. From Fig 12A and 12C, we observed that Ki67 WSIs in borderline ODX scores (i.e., close to the threshold 25) are misclassified more often than WSIs in other ODX score ranges. We also find that predicting the Ki67 WSIs from patients in grade 2 received a higher error rate than other grades. However, as per Fig 12B and 12D, these observations don’t hold in H&E WSIs predictions. Furthermore, our method with intelligent sampling is more computationally efficient in comparison to no sampling. With sampling, the method can make a prediction about 50 times faster than the method without sampling, making it practical to deploy in limited computational settings (see Fig 13).

Our study had some limitations. First, we assigned slide-level labels for all corresponding patches as supervision for the training of patch-level CNN-scorer. This weak supervision strategy is limited when the informative regions constitute only a small portion of the whole slide. With the fast development of self-supervised learning, especially contrastive learning [41, 43, 44], deep learning models can learn the differences between the data without the supervision of annotations. Second, the proposed method could adapt more extreme data by utilizing these new technologies. Secondly, although interpretable, our heatmap visualizations haven’t been analyzed by pathologists. However, its interpretability can make further biomarker investigation and verification reachable. Related research can be even boosted if we make our model a web-based tool since our model is light weighted and easy to implement. Last but not least, the proposed method’s performance on H&E-stained slides is lower than that on Ki67-stained slides. In the current clinical practice, the H&E is still the most routinely used stain for oncological analysis, so an accurate prediction model for H&E-stained slides will have broader application opportunities. Our future work will improve our model’s performance on H&E-stained slides and test our method on a larger independent dataset collected from multiple institutions to reflect the variations in slide preparation and patient characteristics.

## Conclusions

In summary, we presented a deep learning-based method, BCR-Net, to automatically predict ODX risk with a minimal requirement for annotations. The proposed method achieves 0.775 AUC on H&E- and 0.811 AUC on Ki67-stained WSIs. In the future, we will further improve our methodology to achieve higher accuracies on independent datasets to be reliable as a reference to assist clinical diagnosis.

## Supporting information

S1 Fig Attention heatmap visualizations of example H&E-stained slides in the high-risk category. Images in the left column are the H&E-stained slides in the high-risk category. Images in the right column are the corresponding attention heatmaps, where each patch on the WSI is assigned with the value of its attention weight yielded by BCR-Net. The heatmaps are contrast-enhanced for visualization purpose. The bright area in the heatmaps correspond to the WSI area that receive high attention weights.
(TIF)

S2 Fig Attention heatmap visualizations of example H&E-stained slides in the low-risk category. Images in the left column are the H&E-stained slides in the low-risk category. Images in the right column are the corresponding attention heatmaps, where each patch on the WSI is assigned with the value of its attention weight yielded by BCR-Net. The heatmaps are contrast-enhanced for visualization purpose. The bright area in the heatmaps correspond to the WSI area that receive high attention weights.
(TIF)

S3 Fig Attention heatmap visualizations of example Ki67-stained slides in the high-risk category. Images in the left column are the Ki67-stained slides in the high-risk category. Images in the right column are the corresponding attention heatmaps, where each patch on the WSI is assigned with the value of its attention weight yielded by BCR-Net. The heatmaps are contrast-enhanced for visualization purpose. The bright area in the heatmaps correspond to the WSI area that receive high attention weights.
(TIF)

S4 Fig Attention heatmap visualizations of example Ki67-stained slides in the low-risk category. Images in the left column are the Ki67-stained slides in the low-risk category. Images in the right column are the corresponding attention heatmaps, where each patch on the WSI is assigned with the value of its attention weight yielded by BCR-Net. The heatmaps are contrast-enhanced for visualization purpose. The bright area in the heatmaps correspond to the WSI area that receive high attention weights.
(TIF)

S1 Data H&E holdout testing AUCs (BCR-Net). (CSV)

S2 Data Ki67 LTOCV validation AUCs (BCR-Net). (CSV)
