# Correction: BCR-Net: A deep learning framework to predict breast cancer recurrence from histopathology images

**DOI:** 10.1371/journal.pone.0315419

**Published:** 2024-12-05

**Authors:** The PLOS ONE staff

There are errors in the Funding statement. The correct Funding statement is as follows: The research was partially funded by a National Institutes of Health Trailblazer award R21EB029493, with PIs Niazi and Segal leading the project. The research was partially funded by a National Institutes of Health grant R21 CA273665 from the NCI, with PI Gurcan leading the project.

The Acknowledgment section is not indicated. The correct Acknowledgment section is as follows: Disclaimer: The authors take full responsibility for the content of this work, and any opinions expressed do not necessarily reflect the official views of the National Institutes of Health.

The publisher apologizes for the errors.
