# Supplementary figures and images for: BCR-Net: A deep learning framework to predict breast cancer recurrence from histopathology images
Source: PLoS One. 2023 Apr 4;18(4):e0283562. doi: 10.1371/journal.pone.0283562 (PMC10072418; doi:10.1371/journal.pone.0283562)

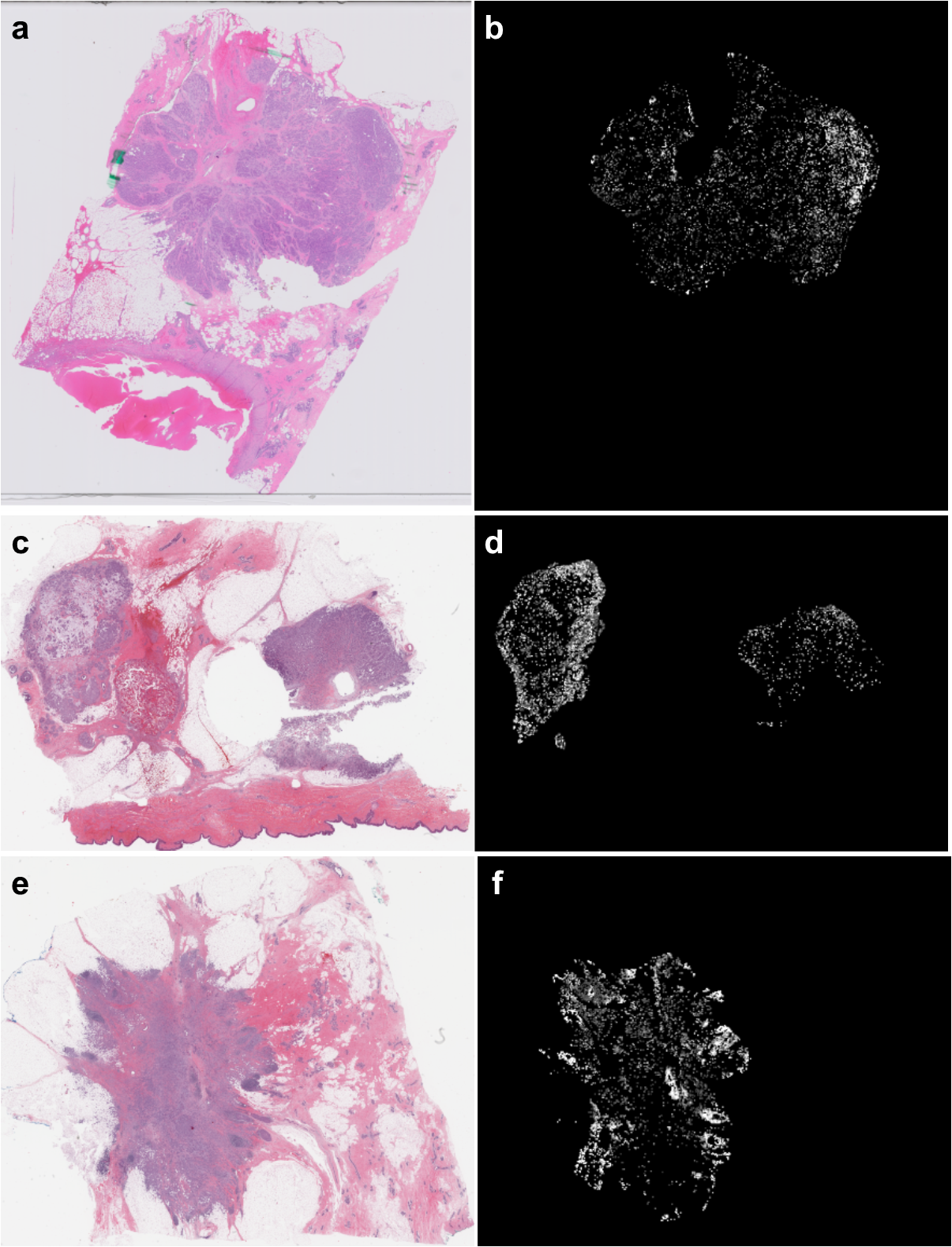

Supplement: S1 Fig — Images in the left column are the H&E-stained slides in the high-risk category. Images in the right column are the corresponding attention heatmaps, where each patch on the WSI is assigned with the value of its attention weight yielded by BCR-Net. The heatmaps are contrast-enhanced for visualization purpose. The bright area in the heatmaps correspond to the WSI area that receive high attention weights. (TIF) [file pone.0283562.s001.tif]

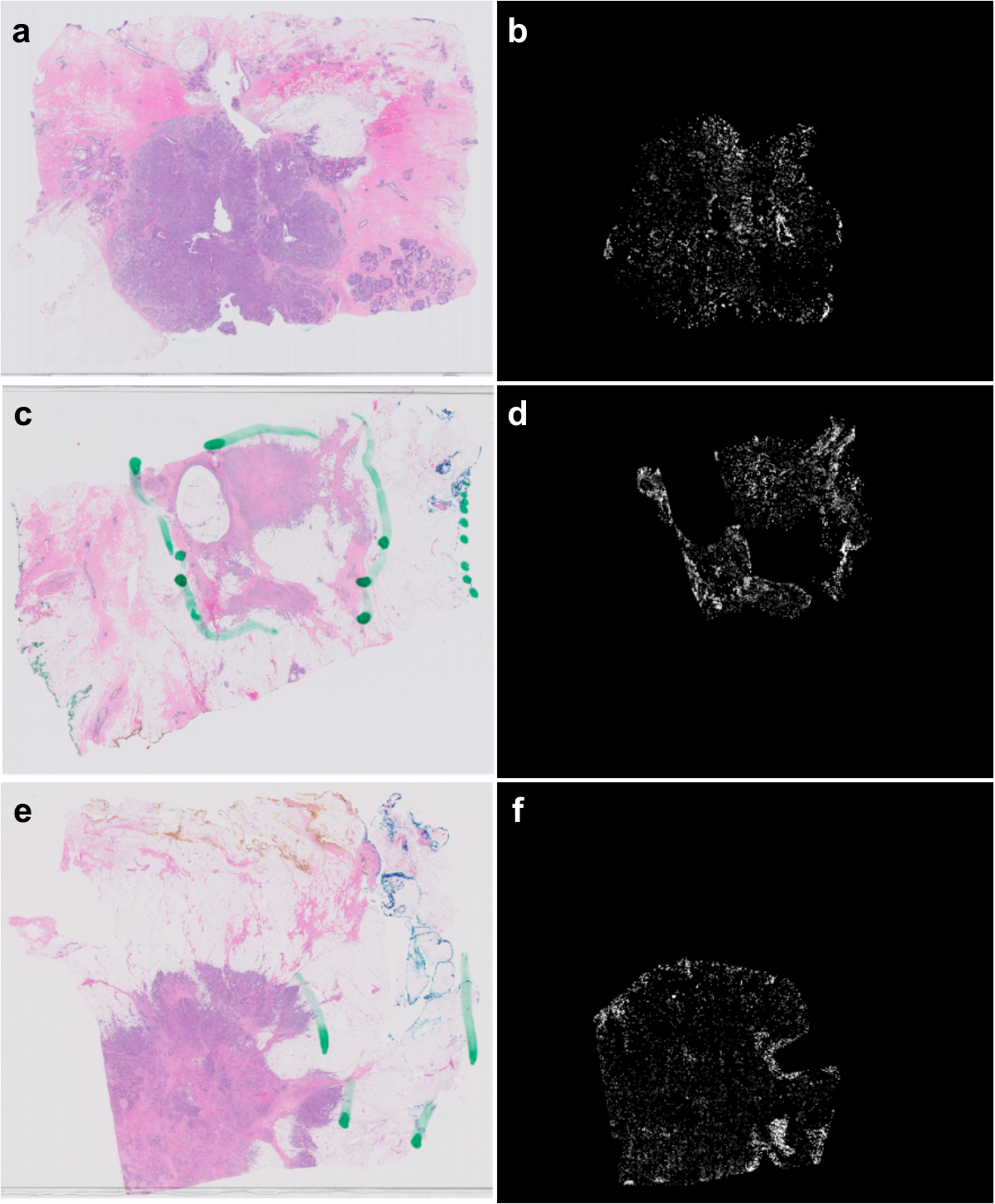

Supplement: S2 Fig — Images in the left column are the H&E-stained slides in the low-risk category. Images in the right column are the corresponding attention heatmaps, where each patch on the WSI is assigned with the value of its attention weight yielded by BCR-Net. The heatmaps are contrast-enhanced for visualization purpose. The bright area in the heatmaps correspond to the WSI area that receive high attention weights. (TIF) [file pone.0283562.s002.tif]

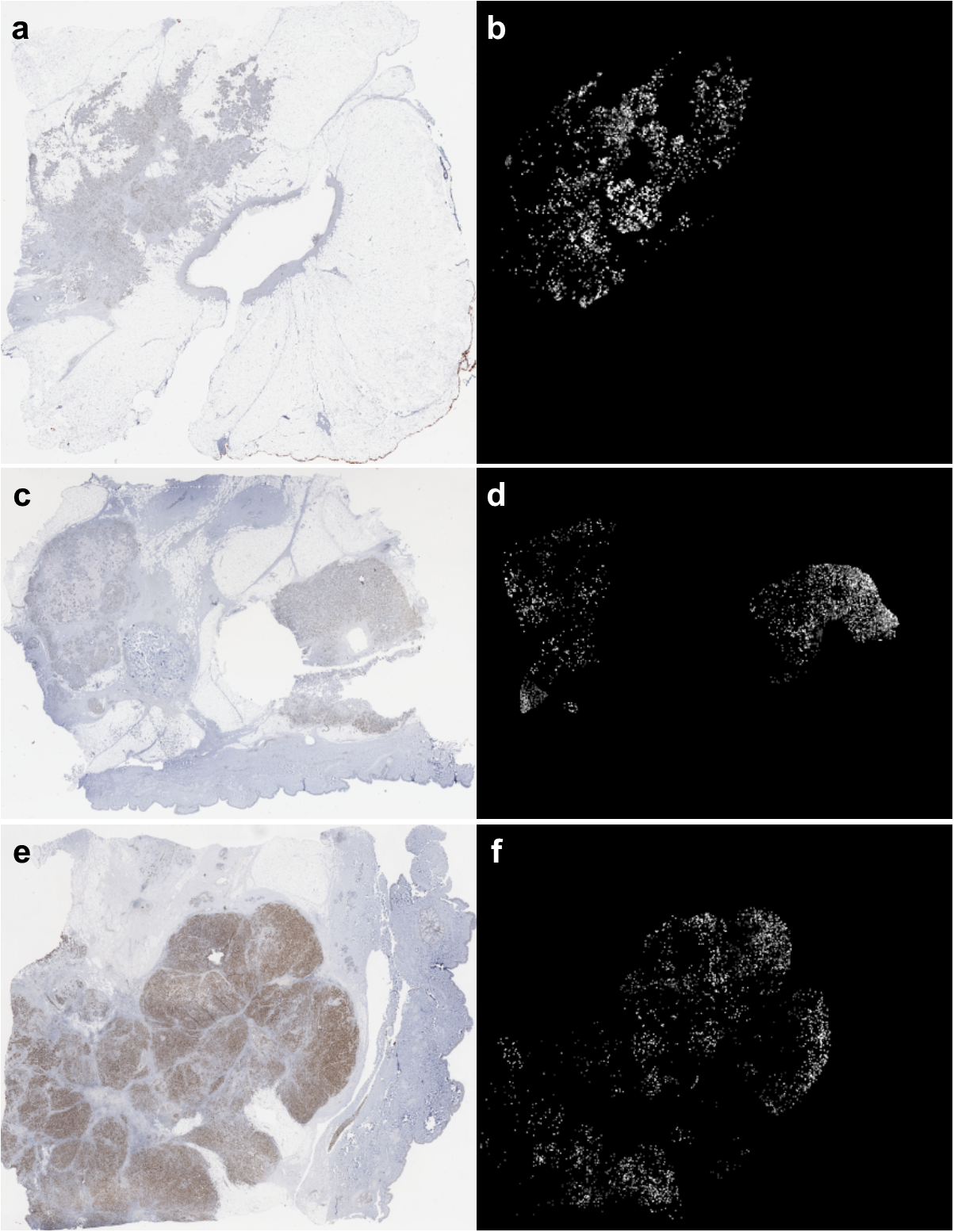

Supplement: S3 Fig — Images in the left column are the Ki67-stained slides in the high-risk category. Images in the right column are the corresponding attention heatmaps, where each patch on the WSI is assigned with the value of its attention weight yielded by BCR-Net. The heatmaps are contrast-enhanced for visualization purpose. The bright area in the heatmaps correspond to the WSI area that receive high attention weights. (TIF) [file pone.0283562.s003.tif]

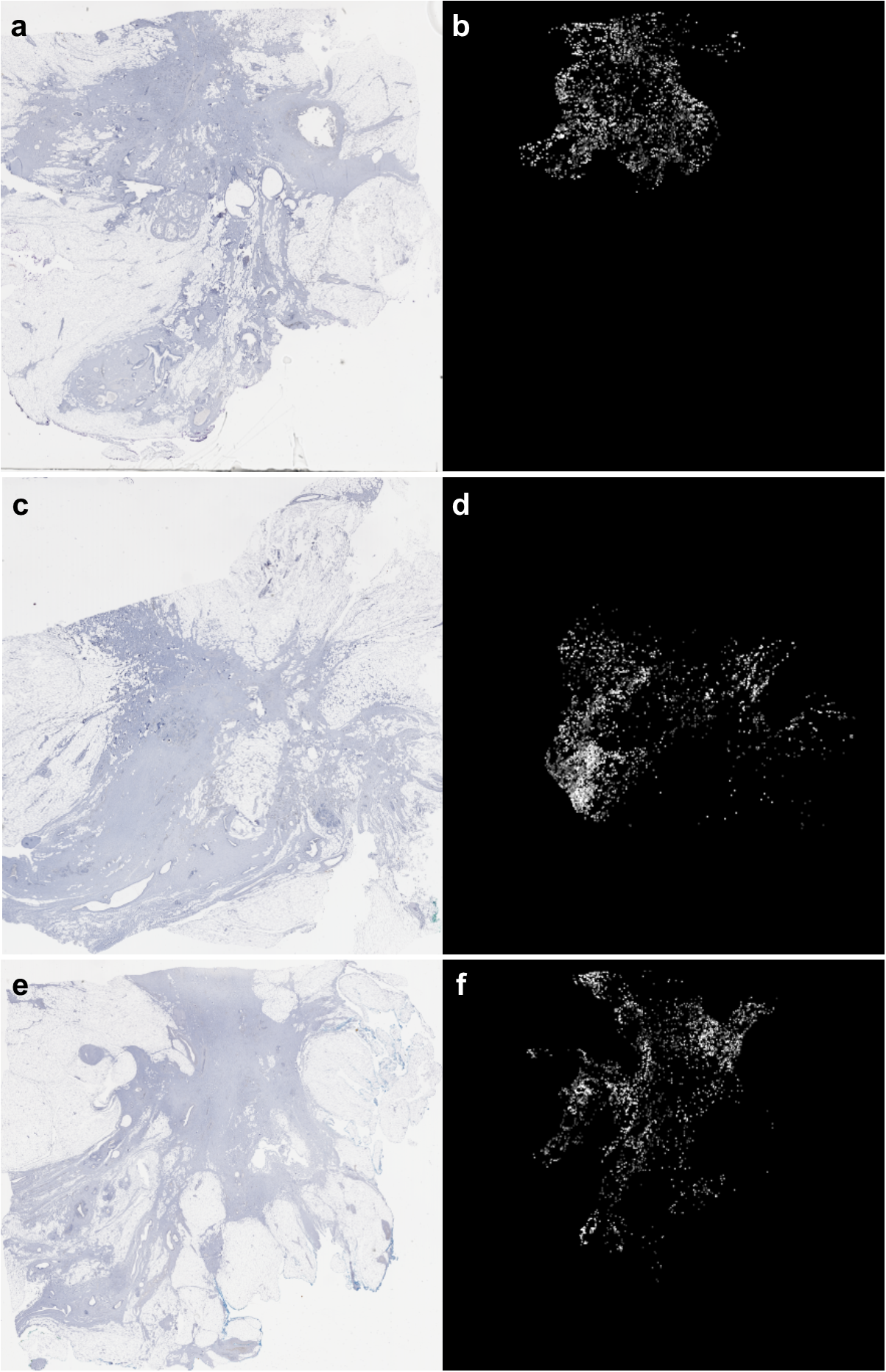

Supplement: S4 Fig — Images in the left column are the Ki67-stained slides in the low-risk category. Images in the right column are the corresponding attention heatmaps, where each patch on the WSI is assigned with the value of its attention weight yielded by BCR-Net. The heatmaps are contrast-enhanced for visualization purpose. The bright area in the heatmaps correspond to the WSI area that receive high attention weights. (TIF) [file pone.0283562.s004.tif]
